# Mineralocorticoid receptor overactivation: targeting systemic impact with non-steroidal mineralocorticoid receptor antagonists

**DOI:** 10.1007/s00125-023-06031-1

**Published:** 2023-12-21

**Authors:** Gianluigi Savarese, Felix Lindberg, Gerasimos Filippatos, Javed Butler, Stefan D. Anker

**Affiliations:** 1 Division of Cardiology, Department of Medicine, Karolinska Institutet, Stockholm, Sweden; 2 Heart and Vascular Theme, Karolinska University Hospital, Stockholm, Sweden; 3 Department of Cardiology, University Hospital Attikon, National and Kapodistrian University of Athens, School of Medicine, Athens, Greece; 4 Baylor Scott and White Research Institute, Dallas, TX USA; 5 Department of Internal Medicine, University of Mississippi, Jackson, MS USA; 6 Department of Cardiology (CVK) and Berlin Institute of Health Center for Regenerative Therapies, German Centre for Cardiovascular Research Partner Site Berlin, Charité Universitätsmedizin, Berlin, Germany; 7 Institute of Heart Diseases, Wroclaw Medical University, Wroclaw, Poland

**Keywords:** Eplerenone, Finerenone, Mineralocorticoid receptor, Mineralocorticoid receptor antagonists, Review, Spironolactone

## Abstract

The overactivation of the mineralocorticoid receptor (MR) promotes pathophysiological processes related to multiple physiological systems, including the heart, vasculature, adipose tissue and kidneys. The inhibition of the MR with classical MR antagonists (MRA) has successfully improved outcomes most evidently in heart failure. However, real and perceived risk of side effects and limited tolerability associated with classical MRA have represented barriers to implementing MRA in settings where they have been already proven efficacious (heart failure with reduced ejection fraction) and studying their potential role in settings where they might be beneficial but where risk of safety events is perceived to be higher (renal disease). Novel non-steroidal MRA have distinct properties that might translate into favourable clinical effects and better safety profiles as compared with MRA currently used in clinical practice. Randomised trials have shown benefits of non-steroidal MRA in a range of clinical contexts, including diabetic kidney disease, hypertension and heart failure. This review provides an overview of the literature on the systemic impact of MR overactivation across organ systems. Moreover, we summarise the evidence from preclinical studies and clinical trials that have set the stage for a potential new paradigm of MR antagonism.

**Graphical Abstract:**

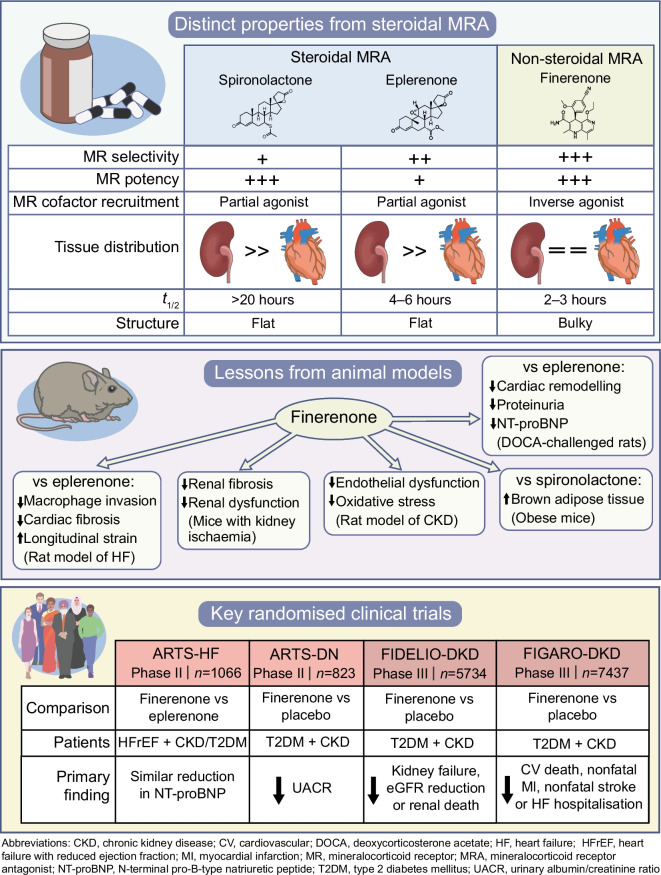

**Supplementary Information:**

The online version of this article contains a slideset of the figures for download available at 10.1007/s00125-023-06031-1.

## Introduction

The mineralocorticoid receptor (MR) plays a key role in human physiology where it regulates fluid, electrolyte and haemodynamic homeostasis. Overactivation of the MR has been demonstrated in individuals with chronic kidney disease (CKD) and individuals with type 2 diabetes mellitus [1], and is associated with increased cardiovascular risk [2–4]. MR overactivation is increasingly recognised to induce inflammation and fibrosis in organ tissues, contributing to CKD and CVD progression, beyond the well-known effects on salt retention and hypertension. The MR has been a pharmacological target for nearly 70 years, although the initial use of MR antagonists (MRA) was primarily for diuretic purposes [5]. In contemporary practice, MRA play important therapeutic roles in resistant hypertension [6], heart failure with reduced ejection fraction (HFrEF) and heart failure with mildly reduced ejection fraction (HFmrEF), where several trials have demonstrated benefits on morbidity and mortality [7–11]. However, real or perceived risk of side effects and tolerability issues associated with traditional steroidal MRA, such as worsening renal function and hyperkalaemia, often represents a barrier to their use in clinical practice [12–15]

Novel non-steroidal MRA have emerged as a promising alternative for targeting MR overactivation, with a better safety profile than traditional steroidal MRA, and demonstrated efficacy in patients with CKD and type 2 diabetes [16–18]. The aim of this review is to provide a comprehensive overview of MR overactivation and the status of non-steroidal MRA, their mechanisms of action, safety profile and therapeutic potential to target the systemic impact of MR overactivation.

## Pathophysiology of MR overactivation

### Historical overview

At the time of its first successful isolation and crystallisation in 1953 [19], aldosterone was primarily recognised as a promotor of renal sodium and fluid retention and potassium excretion [20]. In subsequent years, the compounds that were initially referred to as ‘aldosterone antagonists’ were increasingly understood to block a receptor (the MR) with binding affinity for not only aldosterone but also cortisol [21–24], and were thus more aptly called MRA. In 1987 an important milestone was reached with the cloning of the MR gene [25]. Today, it is accepted that mechanisms independent of aldosterone can contribute to MR activation, and that the role of the MR in disease progression goes beyond its well-known effects on salt and fluid homeostasis, and involves metabolic, proinflammatory and pro-fibrotic pathways [5, 24, 26–28].

### Properties of the MR

The MR belongs to the steroid hormone intracellular receptor family [25, 26]. In its inactivated state, the MR is typically located in the cytoplasm. Its activation following the binding with its steroidal ligands (in human physiology these are mainly aldosterone and cortisol) facilitates its translocation into the cellular nucleus, where it forms complexes with a wide range of cofactors to regulate transcription [29, 30]. However, the MR also exhibits additional effects through pathways that are independent of gene transcription [30–34]. The MR is expressed in multiple organs throughout the body [26], including the kidneys [35–37], heart [36, 38–40], vasculature [36, 38, 41, 42], gastrointestinal tract [36], adipose tissue [43, 44] and central nervous system [36, 45]. The MR binds with similar affinity to cortisol and aldosterone in vitro, but its predominant ligands and functions in vivo are context-dependent according to its location in the body [26, 29, 30, 36, 46].

### Renal and extra-renal effects of MR activation

#### Role of the MR in the kidney

In the kidney, the MR is classically characterised as a regulator of salt and fluid homeostasis [47]. This pathway begins with the production and secretion of aldosterone in the adrenal glands, mediated by activation of the renin–angiotensin system (RAS) in response to hyperkalaemia and hyponatraemia [47]. In renal epithelial cells, the enzyme 11β-hydroxysteroid dehydrogenase (11β-HSD2) converts cortisol into cortisone, which does not bind to the MR, rendering aldosterone its primary ligand [46, 48]. In the distal nephron, MR activation by aldosterone promotes transcription and activity of the epithelial sodium channel (ENaC), resulting in increased sodium and fluid retention and potassium excretion [30, 47]. While historically this is the most-attributed function of the renal MR, evidence from animal models implicates MR activation as an inductor of oxidative stress in the kidney [37], and a key mediator of renal inflammation and fibrosis [28, 49–52].

#### Role of the MR in the heart, immune cells, adipose tissue and vasculature

**Fig. 1:**
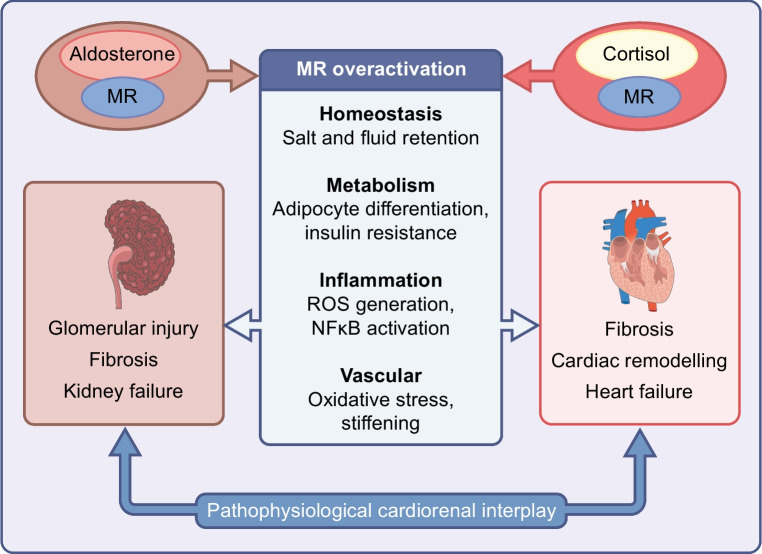
Role of MR overactivation in cardiorenal disease. ROS, reactive oxygen species. This figure is available as part of a downloadable slideset

The potential systemic implications of MR overactivation become apparent when considering the diverse roles of the MR in organs other than the kidneys (Fig. 1) [26]. In cardiomyocytes, where the expression of the MR is not accompanied by the cortisol-converting enzyme 11β-HSD2 [46, 48], cortisol may be a more predominant ligand for the MR. The first suggestion of MR activation as a promotor of cardiac fibrosis originated from the work of Selye in 1958 [53], where the administration of a mineralocorticoid agent in dogs resulted in cardiac necrosis and subsequent fibrotic scarring. Similar findings were later reported in studies of rodents [54, 55]. Immune cell MR activity may play a role in this phenomenon; in mice, macrophage-specific deletion of MR protected against deoxycorticosterone/salt-induced cardiac fibrosis [56]. More recently, knockout of MR in cardiomyocytes and T cells has been shown to improve post-myocardial infarction ventricular remodelling [39, 57]. Insulin resistance, inflammation and adipocyte dysfunction improve with MR blockade in mice [58–61]. Human adipocytes express MRs and have the capacity for aldosterone production [62, 63]. The link between MR activation and insulin resistance has been reported in individuals with primary aldosteronism [64, 65], hypertension [66], CKD [67] and heart failure [63, 68]. In the hypothalamus, MR activation may increase sympathetic drive [69]. The activation of MRs in vascular smooth muscle cells may contribute to vascular oxidative stress, ageing and stiffening [70–72].

#### Mechanisms of MR overactivation

MR overactivation may arise from both aldosterone-mediated and aldosterone-independent pathways, including by cortisol-ligand activation in cells deficient of the cortisol-converting enzyme 11β-HSD2, such as cardiomyocytes [46, 48, 73]. The relationship between falling GFR and increasing aldosterone levels may predispose individuals with CKD to MR overactivation [74]. Treatment with RAS-inhibitors, which is recommended in CKD, hypertension and heart failure, may contribute to long-term elevated aldosterone levels known as ‘aldosterone breakthrough’ or ‘aldosterone escape’ in 30–40% of patients [50, 75–80]. Moreover, adipocyte aldosterone production may contribute to increased MR activation in obese individuals [62, 81], which may be reversed by weight loss [82].

## Therapeutic targeting of MR overactivation

### Steroidal MRA

#### Steroidal MRA in heart failure

Following the development of the first steroidal MRA in the 1950s [83, 84], spironolactone became available in 1960 primarily as a diuretic in patients with oedema, primary aldosteronism and essential hypertension [24, 85]. In recent decades, the indications of steroidal MRA have broadened, reflecting the wider implications of MR inhibition, with perhaps their most far-reaching impact to date in heart failure [7]. In 1999, the RALES RCT demonstrated a 30% reduction in mortality and a 35% reduction in risk of hospitalisation due to heart failure with spironolactone vs placebo in 1663 patients with severe HFrEF [9]. Following RALES, several landmark trials have established the efficacy of the steroidal MRA spironolactone and eplerenone in reducing morbidity and mortality in patients with HFrEF or HFmrEF (Table 1) [8–11].

**Table 1:** Key RCTs on steroidal and non-steroidal MRA in CV and renal disease

Trial	Design	Comparison	Key selection criteria	Key findings
HFrEF				
Steroidal MRA				
RALES (*n*=1663) [9]	RCT, Phase III, double-blinded	Spironolactone vs placebo	HF, NYHA III–IV, EF ≤35%. Serum creatinine ≤221 μmol/l, serum K+ ≤5.0 mmol/l.	Efficacy: Spironolactone reduced all-cause mortality (RR 0.70, 95% CI 0.60, 0.82). Safety: K+ ≥6.0 mmol/l occurred in 2% (spironolactone) vs 1% (placebo).
EMPHASIS-HF (*n*=2737) [8]	RCT, Phase III, double-blinded	Eplerenone vs placebo	HF, NYHA II, EF ≤30% (or 30–30% if QRS duration >130 ms). eGFR ≥30 ml/min per 1.73 m^2^, serum K+ ≤5.0 mmol/l.	Efficacy: Eplerenone reduced the primary composite of CV death or HF hospitalisation (HR 0.63, 95% CI 0.54, 0.74). Safety: K+ ≥5.5 mmol/l occurred in 11.8% (eplerenone) vs 7.2% (placebo).
Non-steroidal MRA				
ARTS-HF (*n*=1066) [127]	RCT, Phase IIb, double-blinded, dose-finding	Finerenone vs eplerenone	HF, EF ≤40%, within 7 days of worsening HF event and co-existing moderate CKD (eGFR 30–60 ml/min per 1.73 m^2^) and/or T2DM. Serum K+ ≤5.0 mmol/l.	Efficacy: There was no difference between finerenone and eplerenone on the primary outcome (percentage of patients with >30% reduction in NT-proBNP at 90 days). The exploratory secondary composite endpoint of all-cause death, CV hospitalisation, or emergency visit for HF was significantly lower (HR 0.56, 95% CI 0.35, 0.90) in the 10–20 mg finerenone group. Safety: K+ ≥5.6 mmol/l occurred in 4.3% overall, with similar incidence across eplerenone and finerenone arms.
HFpEF				
Steroidal MRA				
Aldo-DHF (*n*=422) [86]	RCT, Phase II, double-blinded	Spironolactone vs placebo	Ambulatory patients with HF, NYHA II–III, EF ≥50% and evidence of diastolic dysfunction. eGFR ≥30 ml/min per 1.73 m^2^, serum K+ <5.1 mmol/l.	Efficacy: Spironolactone improved E/e' (adjusted mean difference vs placebo, −1.5, 95% CI −2.0, −0.9) but did not affect peak VO_2_ (co-primary endpoints). Safety: K+ >5.5 mmol/l occurred in 2% (spironolactone) vs 1% (placebo).
TOPCAT (*n*=3445) [87]	RCT, Phase III, double-blinded	Spironolactone vs placebo	Symptomatic HF, EF ≥45%. eGFR ≥30 ml/min per 1.73 m^2^, serum K+ <5.0 mmol/l.	Efficacy: No difference in the primary composite of CV death, aborted cardiac arrest or HF hospitalisation (HR 0.89, 95% CI 0.77, 1.04). The exploratory secondary outcome of HF hospitalisations were lower in the spironolactone arm (HR 0.83, 95% CI 0.69, 0.99). Safety: K+ ≥5.5 mmol/l occurred in 18.7% (spironolactone) vs 9.1% (placebo).
Post-MI				
Steroidal MRA				
EPHESUS (*n*=6642) [10]	RCT, Phase III, double-blinded	Eplerenone vs placebo	Within 3 to 14 days of acute MI (ST-elevation or non-ST-elevation), as well as HF, EF ≤40%. Serum creatinine ≤220 μmol/l, serum K+ ≤5.0 mmol/l.	Efficacy: Eplerenone reduced all-cause mortality (RR 0.85, 95% CI 0.75, 0.96). Safety: K+ ≥6.0 mmol/l occurred in 5.5% (eplerenone) vs 3.9% (placebo).
ALBATROSS (*n*=1603) [143]	RCT, Phase III, open label	Spironolactone open label	Within 72 h of acute MI (ST-elevation or non-ST-elevation) irrespective of EF. eGFR ≥30 ml/min per 1.73 m^2^, serum K+ ≤5.5 mmol/l.	Efficacy: No difference in the primary composite of death, resuscitated cardiac arrest, significant ventricular arrhythmia, indication for implantable defibrillator, or new or worsening HF at 6 month follow-up (HR 0.97, 95% CI 0.73, 1.28). Safety: K+ >5.5 mmol/l occurred in 3% (spironolactone) vs 0.5% (standard of care).
REMINDER (*n*=1012) [144]	RCT, double-blinded	Eplerenone vs placebo	Within 24 h of ST-elevation MI without prior history of HF or previously known EF <40%. eGFR >30 ml/min per 1.73m^2^.	Efficacy: The primary composite (CV mortality, re-hospitalisation or extended initial hospital stay due to HF, sustained ventricular tachycardia/fibrillation, EF ≤40%, or elevated natriuretic peptides at ≥1 month) was lowered by eplerenone (HR 0.58, 95% CI 0.45, 0.76). Safety: K+ >5.5 mmol/l occurred in 5.6% (eplerenone) vs 3.2% (placebo).
Hypertension				
Steroidal MRA				
PATHWAY-2 (*n*=335) [6]	RCT, double-blinded, cross-over design	Spironolactone vs placebo, bisoprolol and doxazosin	Seated clinic systolic BP ≥140 mmHg (or ≥135 mmHg in diabetes) and home systolic BP ≥130 mmHg, despite treatment with maximally tolerated doses of a RAS inhibitor, a CCB and a diuretic. eGFR ≥30 ml/min per 1.73 m^2^, serum K+ within normal range.	Efficacy: Spironolactone was superior in lowering BP vs placebo (–8.70 mmHg, 95% CI −9.72, −7.69), bisoprolol (–4.48, 95% CI –5.50, −3.46), and doxazosin (–4.03, 95% CI –5.04, −3.02). Safety: K+ increased 0.43 mmol/l but with no cases of incident hyperkalaemia in the spironolactone arm.
Non-steroidal MRA				
ESAX-HTN (*n*=1001) [135]	RCT, Phase III, double-blinded, dose-finding	Esaxerenone vs eplerenone	Patients with essential hypertension (systolic/diastolic BP 140–179/90–109). eGFR ≥60 ml/min per 1.73 m^2^, serum K+ <5.1 mmol/l.	Efficacy: The primary endpoints (changes in sitting systolic or diastolic BP at 12 weeks) vs eplerenone 50 mg were unchanged by esaxerenone 2.5 mg but improved by esaxerenone 5 mg. Safety: K+ ≥5.5 mmol/l twice consecutively occurred in 0.6% (esaxerenone 2.5 mg) vs 0.3% (esaxerenone 5 mg) vs 0% (eplerenone 50 mg).
CKD				
Non-steroidal MRA				
ARTS-DN (*n*=823) [128]	RCT, Phase IIb, double-blinded, dose-finding	Finerenone vs placebo	T2DM, UACR ≥3.39 mg/mmol (30 mg/g) and an eGFR >30 ml/min per 1.73 m^2^; treated with at least the minimum recommended dosage of an RAS blocker prior to the screening. Serum K+ ≤4.8 mmol/l.	Efficacy: There was a dose-dependent improvement in the primary outcome (ratio of UACR at day 90 vs baseline) vs placebo at finerenone doses 7.5–20 mg/day, ranging from 21% reduction in the 7.5 mg finerenone group to 38% reduction in the 20 mg finerenone group. Safety: K+ ≥5.6 mmol/l occurred in 0% (placebo), 2.1% (finerenone 1.25 mg), 1.1% (finerenone 2.5 mg), 1.0% (finerenone 5 mg), 2.1% (finerenone 7.5 mg), 3.2% (finerenone 1.5 mg) and 1.7% (finerenone 20 mg).
FIDELIO-DKD (*n*=5734) [16]	RCT, Phase III, double-blinded	Finerenone vs placebo	T2DM, CKD (either: (1) persistent UACR 3.39-33.9 mg/mmol (30–300 mg/g), eGFR 25–60 ml/min per 1.73 m^2^ and history of diabetic retinopathy; or (2) persistent UACR 33.9-565 mg/mmol (300–5000 mg/g) and eGFR 25–75 ml/min per 1.73 m^2^), RAS blocker therapy, serum potassium ≤4.8 mmol/l, no diagnosis of symptomatic chronic HFrEF.	Efficacy: Finerenone reduced risk of the primary composite of kidney failure (eGFR <15 ml/min per 1.73 m^2^, long-term dialysis or kidney transplantation), a sustained >40% reduction in eGFR or death from renal causes (HR 0.82, 95% CI 0.73, 0.93). Safety: Treatment discontinuation due to hyperkalaemia occurred in 2.3% (finerenone) vs 0.9% (placebo).
FIGARO-DKD (*n*=7437) [17]	RCT, Phase III, double-blinded	Finerenone vs placebo	T2DM, CKD (either: (1) persistent UACR 3.39-33.9 mg/mmol (30–300 mg/g), eGFR 25–90 ml/min per 1.73 m^2^; or (2) persistent UACR 33.9-565 mg/mmol (300–5000 mg/g) and eGFR ≥60 ml/min per 1.73 m^2^), RAS blocker therapy, serum potassium ≤4.8 mmol/l, no diagnosis of symptomatic chronic HFrEF.	Efficacy: Finerenone reduced risk of the primary composite of CV death, nonfatal MI, nonfatal stroke or HF hospitalisation (HR 0.87; 95% CI 0.76, 0.98). Safety: Treatment discontinuation due to hyperkalaemia occurred in 1.2% (finerenone) vs 0.4% (placebo).
ESAX-DN [136]	RCT, Phase III, double-blinded	Esaxerenone vs placebo	T2DM, hypertension, RAS blocker therapy, UACR 5.09-33.79 mg/mmol (45–299 mg/g), eGFR ≥30 ml/min per 1.73 m^2^). Serum K+ ≥3.5 and <5.0 mmol/l.	Efficacy: The primary outcome (the proportion of patients achieving UACR remission) was lowered by esaxerone (absolute difference 18%; 95% CI 12%, 25%). Safety: K+ ≥6.0 mmol/l or ≥5.5 mmol/l twice consecutively occurred in 9% (esaxerenone) vs 2% (placebo).
End-stage renal disease				
Steroidal MRA				
DOHAS (*n*=309) [112]	RCT, open label	Spironolactone open label	4-h-long HD 3 times/week for 4 years, serum potassium <6.5 mmol/l, 24 h urinary output <500 ml.	Efficacy: The primary composite of CCV deaths or CCV hospitalisations was lowered by spironolactone (HR 0.404, 95% CI 0.202, 0.809). Safety: Serious hyperkalaemia led to discontinuation in three patients (1.9%) in the spironolactone arm.
Spin-D (*n*=129) [113]	RCT, double-blinded, multiple dosage	Spironolactone vs placebo	Maintenance HD for ≥6 months (or for 3–6 months if there were no changes in target dry weight in past 2 weeks and no hospitalisations in past 6 weeks), serum potassium <6.5 mmol/l.	Safety: The primary safety endpoints of hyperkalaemia (potassium >6.5 mmol/l) and hypotension events were similar with placebo and the overall spironolactone arm, but hyperkalaemia events increased with the spironolactone dose (0.50, 0.32, 0.23 and 0.89 events per patient-year in the placebo, 12.5 mg, 25 mg and 50 mg groups, respectively).
MiREnDa (*n*=97) [114]	RCT, double-blinded	Spironolactone vs placebo	Maintenance HD, no MRA treatment within the last 6 months, no history of hyperkalaemia (potassium ≥6.5 mmol/l).	Efficacy: There was no difference in the primary efficacy endpoint of change in LVMi. Safety: Moderate hyperkalaemia episodes (6.0–6.5 mmol/l), but not severe (≥6.5 mmol/l), occurred more frequently with spironolactone (155 vs 80 events, *p*=0.034).

CCB, calcium channel blocker; CCV, cerebral or cardiovascular; CV, cardiovascular; EF, ejection fraction; HD, haemodialysis; HF, heart failure; LVMi, left ventricular mass index; MI, myocardial infarction; NT-proBNP, N-terminal pro-B-type natriuretic peptide; T2DM, type 2 diabetes mellitus

The findings in HFrEF/HFmrEF have not been convincingly translated to patients with heart failure and preserved ejection fraction (HFpEF). The Aldo-DHF trial randomised 422 patients with chronic New York Heart Association (NYHA) class II–III heart failure with ejection fraction ≥50% and diastolic dysfunction to receive spironolactone or placebo. Spironolactone improved diastolic function, but had no effect on exercise capacity, symptoms or quality of life [86]. The subsequent larger TOPCAT trial randomised 3445 patients with symptomatic heart failure and an ejection fraction ≥45% to receive spironolactone or placebo, and barely missed its primary composite endpoint of cardiovascular death, aborted cardiac arrest and heart hospitalisation due to heart failure [87]. The near-miss in TOPCAT was met with debate concerning methodological and conduct issues, leaving many to consider the question of steroidal MRA in HFpEF yet unanswered [88]. Two ongoing RCTs aim to resolve the question of steroidal MRA in HFpEF (SPIRIT-HF: NCT04727073; SPIRRIT-HFpEF: NCT02901184) (Table 2) [89, 90].

**Table 2:** Key ongoing and awaited RCTs targeting MR/aldosterone pathways in cardiovascular and renal disease

RCT, NCT number	Description (estimated enrolment)	Projected read-out
Steroidal MRA		
SPIRIT-HF, NCT04727073	Double-blind RCT comparing spironolactone against placebo in HF and EF ≥40% (*n*=1300)	2024
ALCHEMIST, NCT01848639	Double-blind RCT comparing spironolactone against placebo in patients on chronic HD (*n*=825)	2024
ACHIEVE, NCT03020303	Double-blind RCT comparing spironolactone against placebo in patients on HD or PD (*n*=2750)	2025
SPIRRIT, NCT02901184	Open label registry-based RCT on spironolactone initiation in patients with HF and EF ≥40% (*n*=2000)	2026
Non-steroidal MRA		
FINEARTS-HF, NCT04435626	Double-blind RCT comparing finerenone against placebo in patients with HF and EF ≥40% (*n*=6016)	2024
CONFIDENCE, NCT05254002	Double-blind RCT comparing finerenone and dapagliflozin against finerenone alone and dapagliflozin alone in patients with DKD (*n*=807)	2024
CLARION-CKD, NCT04968184	Double-blind RCT comparing KBP-5074 (a novel non-steroidal MRA) against placebo in patients with uncontrolled hypertension and CKD stages 3b/4 (*n*=600)	2025
FIND-CKD, NCT05047263	Double-blind RCT comparing finerenone against placebo in patients with non-diabetic CKD (*n*=1574)	2026
FIONA, NCT05196035	Double-blind RCT comparing finerenone against placebo in children (age 6 months to 17 years) with CKD (*n*=219)	2027
Non-steroidal MR modulators		
MIRACLE, NCT04595370	Double-blind dose-finding RCT comparing balcinrenone (AZD9977, a novel non-steroidal MR modulator) in different doses together with daplagliflozin against dapagliflozin alone in patients with HF, EF <60% and CKD (eGFR 20–60 ml/min per 1.73m^2^; *n*=147)	2023
Aldosterone synthase inhibitors		
NCT05182840	Double-blind dose-finding RCT comparing BI 690517 (a novel aldosterone synthase inhibitor) with or without empagliflozin against placebo in patients with CKD (*n*=714)	2023

CV, cardiovascular; DKD, diabetic kidney disease; EF, ejection fraction; HD, haemodialysis; PD, peritoneal dialysis; HF, heart failure; NCT number, ClinicalTrials.gov identifier; T2DM, type 2 diabetes mellitus

#### Side-effect profile and barriers to implementation of steroidal MRA in heart failure

Spironolactone is structurally similar to progesterone and is a potent but unspecific antagonist of the MR [91]. Accordingly, gynaecomastia and breast pain have been known side effects of spironolactone since the 1960s [92]. Such side effects occurred in 10% vs 1% the spironolactone vs placebo arms of RALES [9]. Compared with spironolactone, eplerenone is a highly specific steroidal MRA, and accordingly has a more favourable anti-androgenic side-effect profile [93]. In the EPHESUS trial, the eplerenone and placebo arms showed similar rates of gynaecomastia and impotence [10]. Another limitation of steroidal MRA is the associated increase in potassium levels and therefore the risk of hyperkalaemic events [94]. The increase in prescriptions of spironolactone following the publication of RALES led to a higher incidence of hyperkalaemia in real-world data [95]. While hyperkalaemia is associated with arrhythmias and mortality [96–98], part of its prognostic impact in heart failure may stem from its link with the discontinuation of treatment with MRA [13, 99]. This hypothesis was behind the rationale for the DIAMOND trial, which demonstrated that the novel potassium-binder patiromer facilitated MRA use in patients with HFrEF with current or previous hyperkalaemia [100]. Despite constituting one of the four pillars of evidence-based pharmacotherapy for HFrEF [7], real and perceived risk of hyperkalaemia-related adverse events associated with steroidal MRA remains a barrier to their widespread implementation in clinical practice. Even mild degrees of CKD, where their efficacy on mortality/morbidity has been proven, are associated with greater underuse [101]. A recently published post hoc analysis of the EMPHASIS-HF and TOPCAT trials reported that MRA use was associated with an approximately 2 ml/min per 1.73 m^2^ initial decline in eGFR during the initial 4–6 months, but was without apparent long-term effects on renal function [102]. Routine care data from different healthcare systems indicate that only 37–40% of patients with HFrEF receive a steroidal MRA [13, 14], and discontinuation of treatment is common [103].

#### Steroidal MRA in CKD

Small trials demonstrating renal benefits of MR blockade in animal models of kidney disease [104, 105], and of steroidal MRA on reducing proteinuria in patients with CKD [106, 107], prompted optimism surrounding their potential use in nephrology [108]. A meta-analysis pooling data from 22 RCTs assessing non-selective steroidal MRA (1441 participants) and six studies assessing selective steroidal MRA (925 participants) in the setting of proteinuric CKD stages I–IV showed beneficial effects on proteinuria but increased risk of hyperkalaemia and acute kidney injury, and uncertain effects on GFR [109]. None of the included studies had a follow-up longer than 12 months. These findings are in overall agreement with previous meta-analyses on steroidal MRA in CKD [110, 111]; thus, there has been insufficient data to estimate effects on hard renal or mortality endpoints.

Few studies have assessed safety and efficacy of steroidal MRA in patients on dialysis. The 2014 Dialysis Outcomes Heart Failure Aldactone Study (DOHAS) randomly assigned spironolactone in 309 patients with oligoanuric haemodialysis, and showed a striking 60% reduction in the primary composite outcome of cardiovascular and cerebrovascular mortality and hospitalisations during the 3 year follow-up [112]. However, the study was limited by an open-label design. Two subsequent RCTs, the Spin-D (*n*=129, 36 week follow-up) and MiREnDa (*n*=97, 40 week follow-up) trials, found that spironolactone compared with placebo resulted in increases in potassium levels and moderate hyperkalaemia events, but no significant differences in severe hyperkalaemia (potassium ≥6.5 mmol/l) [113, 114]. While these trials suggested spironolactone to be reasonably safe provided there is stringent monitoring, there were no effects on left ventricular mass index or function. A Cochrane meta-analysis summarised the evidence from 16 RCTs including a total of 1446 patients with CKD requiring dialysis, suggesting that spironolactone likely reduces cardiovascular and all-cause mortality in this context (RR 0.37; 95% CI 0.22, 0.64) but somehow with an increase in risk of hyperkalaemia (RR 1.41; 95% CI 0.72, 2.78) [115]. The ongoing ALCHEMIST (NCT01848639) and ACHIEVE (NCT00277693) trials aim to further establish the safety and efficacy of steroidal MRA in patients undergoing haemodialysis.

### Non-steroidal MRA

The clearly demonstrated clinical benefits of steroidal MRA, together with their limited use due to their actual or perceived safety profile, stimulated research aiming to inhibit the MR while maintaining a better safety profile. Among non-steroidal compounds, dihydropyridine-based antagonists displayed high MR potency and selectivity. The dihydropyridine-derivative BAY 94–8862 (today known as finerenone) was identified as a novel non-steroidal MRA that at least matched spironolactone in its potency, while displaying unprecedented selectivity to the MR in vitro, and promising efficacy vs eplerenone in preclinical animal models in vivo [116].

#### Distinct characteristics of non-steroidal MRA

**Fig. 2:**
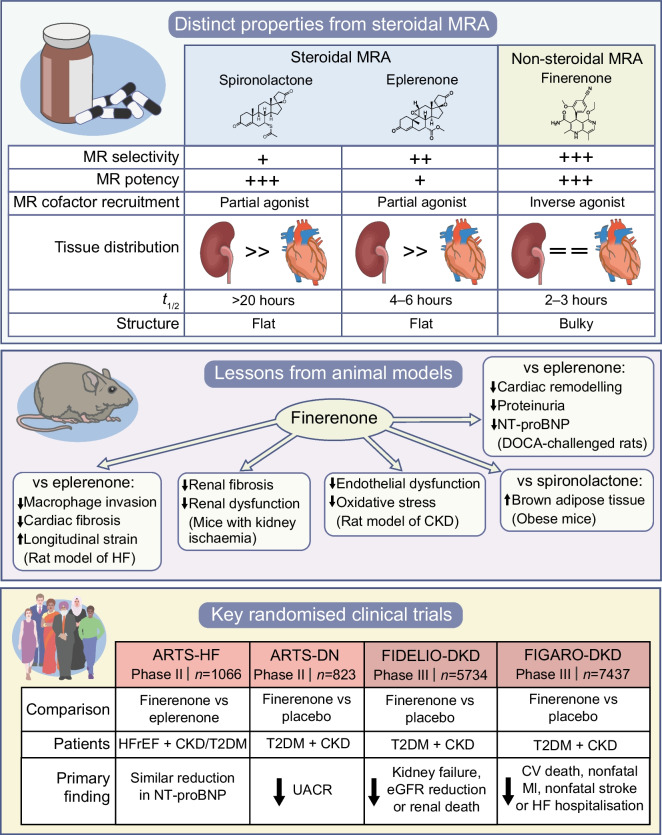
Distinct characteristics and mechanisms of finerenone vs classical steroidal MRA. CV, cardiovascular; DOCA, deoxycorticosterone acetate; HF, heart failure; MI, myocardial infarction; NT-proBNP, N-terminal pro-B-type natriuretic peptide; T2DM, type 2 diabetes mellitus. This figure is available as part of a downloadable slideset

Beyond its MR-selectivity and potency, finerenone carries several attributes that could lead to different clinical effects as compared with spironolactone and eplerenone (Fig. 2). First, upon binding the MR, steroidal MRA convey partial agonism on the MR cofactor recruitment. Since this partial agonism is less potent than the one exerted by aldosterone, spironolactone and eplerenone exhibit an antagonistic effect that is mainly apparent in the presence of aldosterone. In contrast, finerenone acts as an inverse agonist ligand, i.e. it reduces MR cofactor recruitment even in the absence of aldosterone [5, 117]. Second, while the tissue distribution of steroidal MRA is more concentrated in the kidneys, finerenone displays a balanced distribution in the heart and the kidneys [118], potentially enhancing the inhibition of proinflammatory and pro-fibrotic effects of cardiac MR activation. Third, the shorter plasma *t*_½_ of finerenone (2–3 h) compared with eplerenone (4–6 h) and spironolactone (long) might translate into a lower risk for hyperkalaemic events [119].

Several studies in animal models support the idea that the distinct properties of finerenone might translate into clinical benefits. Finerenone improved cardiac remodelling, natriuretic peptide concentrations and proteinuria to a greater degree than eplerenone in deoxycorticosterone acetate-/salt-challenged rats [118]. Finerenone, but not eplerenone, also improved systolic and diastolic function and reduced natriuretic peptides in rats with heart failure induced by coronary artery ligation [118]. In a mouse model of cardiac fibrosis, finerenone, but not eplerenone, inhibited macrophage invasion and improved cardiac fibrosis. It also improved global longitudinal peak strain more than eplerenone [120]. In mice exposed to kidney ischaemia, finerenone protected against subsequent renal fibrosis and dysfunction [121, 122]. In a model of CKD induced by endothelial dysfunction, reduced oxidative stress appeared to mediate the effect of finerenone on improved endothelial dysfunction [123]. One recent study on obese mice showed that finerenone, but not spironolactone, enhanced the activation of brown adipose tissue [124]. Interestingly, in a model of hypertension-induced end-organ damage, a synergistic effect between the sodium–glucose cotransporter 2 inhibitor (SGLT2i) empagliflozin and finerenone was observed in the improvement of proteinuria, serum creatinine levels, histopathological signs of cardiac and renal lesions, and mortality [125].

### Randomised evidence on non-steroidal MRA

#### Phase II trials

Following promising preclinical results, finerenone was investigated in the MR Antagonist Tolerability Study (ARTS) Phase II RCT. ARTS part B enrolled 392 patients with HFrEF (ejection fraction ≤40%), NYHA class II–III and an eGFR 30–60 ml/min per 1.73 m^2^, and randomised the participants to different doses of finerenone or placebo and active treatment with spironolactone. During the 4 week follow-up, hyperkalaemia occurred less frequently in the finerenone (5.3%) than the spironolactone arm (12.7%), as did renal impairment (3.8% vs 28.6%). Finerenone and spironolactone demonstrated similar improvements in natriuretic peptides and albuminuria [126].

ARTS was followed by the larger ARTS-HF multicentre Phase IIb dose-finding study comparing finerenone with eplerenone in patients with worsening HFrEF and concomitant moderate CKD and/or type 2 diabetes (i.e. eGFR >30 ml/min per 1.73 m^2^ in type 2 diabetes or 30–60 ml/min per 1.73 m^2^ without type 2 diabetes). In 1066 randomised patients, during 90 days follow-up the eplerenone and finerenone dose groups showed similar frequency of the primary outcome (the percentage of patients with >30% reduction in N-terminal pro-B-type natriuretic peptide [NT-proBNP] at 90 days), as well as of the key safety endpoint of hyperkalaemia (4.7% incidence in the eplerenone arm and 3.6–6.3% in the finerenone dose arms). Despite the short 90 day follow-up, the exploratory secondary composite endpoint of all-cause death, cardiovascular hospitalisation or emergency visit for heart failure was significantly lower (HR 0.56; 95% CI 0.35, 0.90) in the group assigned to finerenone 10 mg followed by up-titration to 20 mg at day 30 vs eplerenone [127].

ARTS-Diabetic Nephropathy (ARTS-DN) compared finerenone at different doses with placebo in 823 patients with type 2 diabetes, albuminuria (urinary albumin/creatinine ratio [UACR] ≥3.39 mg/mmol [30 mg/g]), eGFR >30 ml/min per 1.73 m^2^ and with a concomitant RAS blocker prior to screening. The trial demonstrated a dose-dependent reduction in UACR with finerenone at the dosages 7.5–20 mg/day, as compared with placebo, ranging from 21% reduction in the 7.5 mg finerenone group to 38% reduction in the 20 mg finerenone group. In the trial, which excluded patients with serum potassium >4.8 mmol/l at screening, finerenone discontinuation due to hyperkalaemia occurred in only 1.8% of patients at doses 7.5–20 mg/day [128].

#### Phase III trials

Two double-blind RCTs were launched as part of the Phase III programme to assess the safety and efficacy of finerenone in CKD with type 2 diabetes in terms of renal (FInerenone in reducing kiDnEy faiLure and dIsease prOgression in DKD [FIDELIO-DKD]) and cardiovascular endpoints (FInerenone in reducinG cArdiovascular moRtality and mOrbidity in DKD [FIGARO-DKD]) [16, 17].

FIDELIO-DKD enrolled 5734 patients with type 2 diabetes and CKD who were treated with a RAS blocker [16]. The CKD inclusion criteria was met if either of the following was fulfilled: (1) persistent moderate albuminuria (UACR 3.39–33.9 mg/mmol [30–300 mg/g]), eGFR 25–60 ml/min per 1.73 m^2^ and history of diabetic retinopathy; or (2) persistent severe albuminuria (UACR 33.9–565 mg/mmol [300–5000 mg/g]), and eGFR 25–75 ml/min per 1.73 m^2^. As in previous trials of the finerenone programme, patients were excluded if serum potassium was >4.8 mmol/l. Another key exclusion criterion was symptomatic chronic HFrEF, which comes with a class 1A recommendation for steroidal MRA [7]. The primary endpoint was the composite of kidney failure (eGFR <15 ml/min per 1.73 m^2^, long-term dialysis or kidney transplantation), a sustained >40% reduction in eGFR, or death from renal causes. Finerenone was associated with an 18% reduction of the primary outcome vs placebo during 2.6 years median follow-up, with consistent effects across patient subgroups. Finerenone also reduced by 14% the pre-specified secondary endpoint of cardiovascular death, nonfatal myocardial infarction, nonfatal stroke or hospitalisation due to heart failure. Hyperkalaemia leading to treatment discontinuation was 2.3% with finerenone and 0.9% with placebo [16].

The purpose of the FIGARO-DKD (*n*=7437) trial was to preferentially examine cardiovascular, rather than renal, outcomes. Accordingly, while other inclusion criteria were similar between FIGARO-DKD and FIDELIO-DKD, the CKD criterion in FIGARO-DKD was less stringent in selecting for renal disease. CKD in FIGARO-DKD was considered fulfilled if either of the following were met: (1) persistent moderate albuminuria (UACR 3.39–33.9 mg/mmol [30–300 mg/g]) and eGFR 25–90 ml/min per 1.73 m^2^; or (2) persistent severe albuminuria (UACR 33.9–565 mg/mmol [300–5000 mg/g]) and eGFR ≥60 ml/min per 1.73 m^2^. The primary composite outcome (cardiovascular death, nonfatal myocardial infarction, nonfatal stroke or hospitalisation due to heart failure) was reduced by 13% in the finerenone arm during 3.4 years median follow-up. Despite the exclusion of patients with HFrEF, a 29% reduction in hospitalisations due to heart failure was the main driver. Similarly, as in the previous finerenone trials, incidence of hyperkalaemia-related discontinuation was low (1.2%) and the rates of adverse events were similar across study arms [17].

The finerenone in CKD and type 2 diabetes: combined FIDELIO-DKD and FIGARO-DKD trial programme analysis (FIDELITY) was a pooled analysis from both trials, including data from 13,026 patients and a median follow-up of 3.0 years. Finerenone reduced risk of the composite cardiovascular outcome (cardiovascular death, nonfatal myocardial infarction, nonfatal stroke or hospitalisation due to heart failure) by 14% and of the composite renal outcome (kidney failure, a sustained ≥57% decrease in eGFR from baseline over ≥4 weeks or renal death) by 23% [18]. The mean 4 month change in UACR was 32% lower with finerenone vs placebo [18]. Benefits with finerenone on heart failure outcomes (first hospitalisation due to heart failure; cardiovascular death or first hospitalisation due to heart failure; recurrent hospitalisations due to heart failure; and cardiovascular death or recurrent hospitalisations due to heart failure) were consistent across eGFR and/or UACR categories [129]. Post hoc analyses from the finerenone Phase III programme have suggested that finerenone might decrease the incidence of heart failure [130] and atrial fibrillation [131]. A pre-specified FIDELIO-DKD subgroup analysis did not detect any effect modification on the composite cardiovascular outcome according to history of heart failure (heart failure: HR 0.73; no heart failure: HR 0.90; *p-*interaction: 0.33) [132]. Importantly, since patients with HFrEF were excluded from the trial, the patients in the heart failure group predominantly had HFpEF, where steroidal MRA have not proven efficacious [87]. The ongoing FINEARTS (NCT04435626) trial will compare finerenone with placebo in 6000 patients with HFmrEF or HFpEF, and is expected to be finalised in 2024. Preclinical models suggested a synergistic effect with concomitant SGLT2i and finerenone use [125]. However, a FIDELITY subgroup analysis did not detect significant interaction with SGLT2i use on the cardiovascular composite (SGLT2i: HR 0.67; no SGLT2i: HR 0.87; *p*-interaction: 0.46) or the renal composite (SGLT2i: HR 0.42; no SGLT2i: HR 0.80; *p*-interaction: 0.29). However, only 6.7% of patients received an SGLT2i at baseline, and 8.5% initiated during the trial [133]. The ongoing CONFIDENCE (NCT05254002) trial will randomly assign 807 patients with type 2 diabetes and CKD to receive SGLT2i and/or finerenone alone or in combination.

Although to date finerenone has been investigated in the largest study programme on non-steroidal MRA, there are other compounds that have been studied in different settings. Esaxerenone is a non-steroidal MRA that has been approved in Japan for the treatment of hypertension and DKD [134]. In the 12 week double-blind Phase III RCT ESAX-HTN, 2.5 mg esaxerenone was noninferior and 5 mg esaxerenone was superior to 50 mg eplerenone in reducing BP in 1001 Japanese patients with essential hypertension, with similar rates of adverse events across study arms [135]. In ESAX-DN, a 52 week double-blind Phase III RCT enrolling 455 patients with DKD on RAS inhibitor therapy, esaxerenone improved albuminuria vs placebo (HR for time to first remission of albuminuria: 5.13; 95% CI 3.27, 8.04) [136]. A Phase II dose-finding RCT (*n*=293) in DKD reported that the non-steroidal MRA apararenone yielded a dose-dependent 37–53% reduction in albuminuria, whereas the placebo arm reported a 14% increase [137].

An emerging possibility to target MR activation with a further improved safety profile may involve MR modulators that do not affect renal potassium excretion. Balcinrenone (AZD9977), an MR modulator that showed organ protection capabilities without affecting urinary sodium/potassium ratio in animal models [138], is currently being evaluated vs placebo in the Phase IIb RCT MIRACLE, enrolling 147 patients with heart failure and CKD (NCT04595370).

Aldosterone also has non-MR-mediated actions, which might not be targetable by the blockage of the MR alone [139], and therefore aldosterone synthase inhibitors might represent another therapeutic opportunity. Baxdrostat is a highly selective aldosterone synthase inhibitor that is being evaluated in resistant hypertension [140]. Although Phase II trials BrigHTN (*n*=248, significant BP lowering effect vs placebo) [141] and HALO (*n*=249, no difference in BP change vs placebo) [142] reported conflicting results, a Phase III trial is planned to start during 2023. Another aldosterone synthase inhibitor, BI 690517, is being evaluated with or without empagliflozin vs placebo for the treatment of CKD, in a Phase II RCT enrolling 714 patients (NCT05182840).

## Conclusion

MR overactivation has deleterious effects on salt and fluid homeostasis, end-organ inflammation, fibrosis and metabolic dysregulation. Classical steroidal MRA have achieved tremendous success in improving outcomes in heart failure, but their adverse effect profile limits their use in clinical practice. Novel non-steroidal MRA have distinct pharmacokinetic and pharmacodynamic properties that potentially translates into favourable clinical effects and better safety profile vs steroidal MRA, and might have the potential to further target the systemic impact of MR overactivation in cardiorenal and metabolic syndromes.

### Supplementary Information

Below is the link to the electronic supplementary material.

Supplementary file1 (PPTX 594 KB)

## Abbreviations

11β-HSD2: 11β-Hydroxysteroid dehydrogenase
CKD: Chronic kidney disease
FIDELIO-DKD: FInerenone in reducing kiDnEy faiLure and dIsease prOgression in DKD
FIDELITY: The finerenone in CKD and type 2 diabetes: combined FIDELIO-DKD and FIGARO-DKD trial programme analysis
FIGARO-DKD: FInerenone in reducinG cArdiovascular moRtality and mOrbidity in DKD
HFrEF: Heart failure with reduced ejection fraction
HFmrEF: Heart failure with mildly reduced ejection fraction
HFpEF: Heart failure with preserved ejection fraction
MR: Mineralocorticoid receptor
MRA: Mineralocorticoid receptor antagonists
NYHA: New York Heart Association
RAS: Renin–angiotensin system
SGLT2i: Sodium–glucose cotransporter 2 inhibitor
UACR: Urinary albumin/creatinine ratio

## Authors’ relationships and activities

GS reports grants and personal fees from Vifor, grants and personal fees from Boehringer Ingelheim, grants and personal fees from AstraZeneca, personal fees from Servier, grants and personal fees from Novartis, grants and personal fees from Cytokinetics, personal fees from Medtronic, grants from Boston Scientific, grants and personal fees from Pharmacosmos, grants from Merck, grants from Bayer, personal fees from TEVA, personal fees from INTAS, personal fees from Abbott, outside the submitted work. FL declares that there are no relationships or activities that might bias, or be perceived to bias, his work. GF reports lecture fees and/or that he is a committee member of trials and registries sponsored by Amgen, Bayer, Boehringer Ingelheim, Medtronic, Novartis, Servier, Impulse Dynamics and Vifor Pharma. He has received research support from the European Union. JB reports personal fees from Abbott, American Regent, Amgen, Applied Therapeutic, AstraZeneca, Bayer, Boehringer Ingelheim, Bristol Myers Squibb, Cardiac Dimension, Cardior, CVRx, Cytokinetics, Edwards, Element Science, Innolife, Impulse Dynamics, Imbria, Inventiva, Lexicon, Lilly, LivaNova, Janssen, Medtronics, Merck, Occlutech, Novartis, Novo Nordisk, Pfizer, Pharmacosmos, Pharmain, Roche, Sequana, SQ Innovation and Vifor. SDA reports grants and personal fees from Vifor and Abbott Vascular, and personal fees for consultancies, trial committee work and/or lectures from Actimed, Amgen, Astra Zeneca, Bayer, Boehringer Ingelheim, Bioventrix, Brahms, Cardiac Dimensions, Cardior, Cordio, CVRx, Cytokinetics, Edwards, Farraday Pharmaceuticals, GSK, HeartKinetics, Impulse Dynamics, Novartis, Occlutech, Pfizer, Repairon, Sensible Medical, Servier, Vectorious and V-Wave. Named co-inventor of two patent applications regarding MR-proANP (DE 102007010834 & DE 102007022367), but he does not benefit personally from the related issued patents.
