# Supplementary material for: Mineralocorticoid receptor overactivation: targeting systemic impact with non-steroidal mineralocorticoid receptor antagonists
Source: Diabetologia. 2023 Dec 21;67(2):246–62. doi: 10.1007/s00125-023-06031-1 (PMC10789668; doi:10.1007/s00125-023-06031-1)
Supplement: Supplementary file 1 — Supplementary file1 (PPTX 594 KB) [file 125_2023_6031_MOESM1_ESM.pptx]

## Slide 1
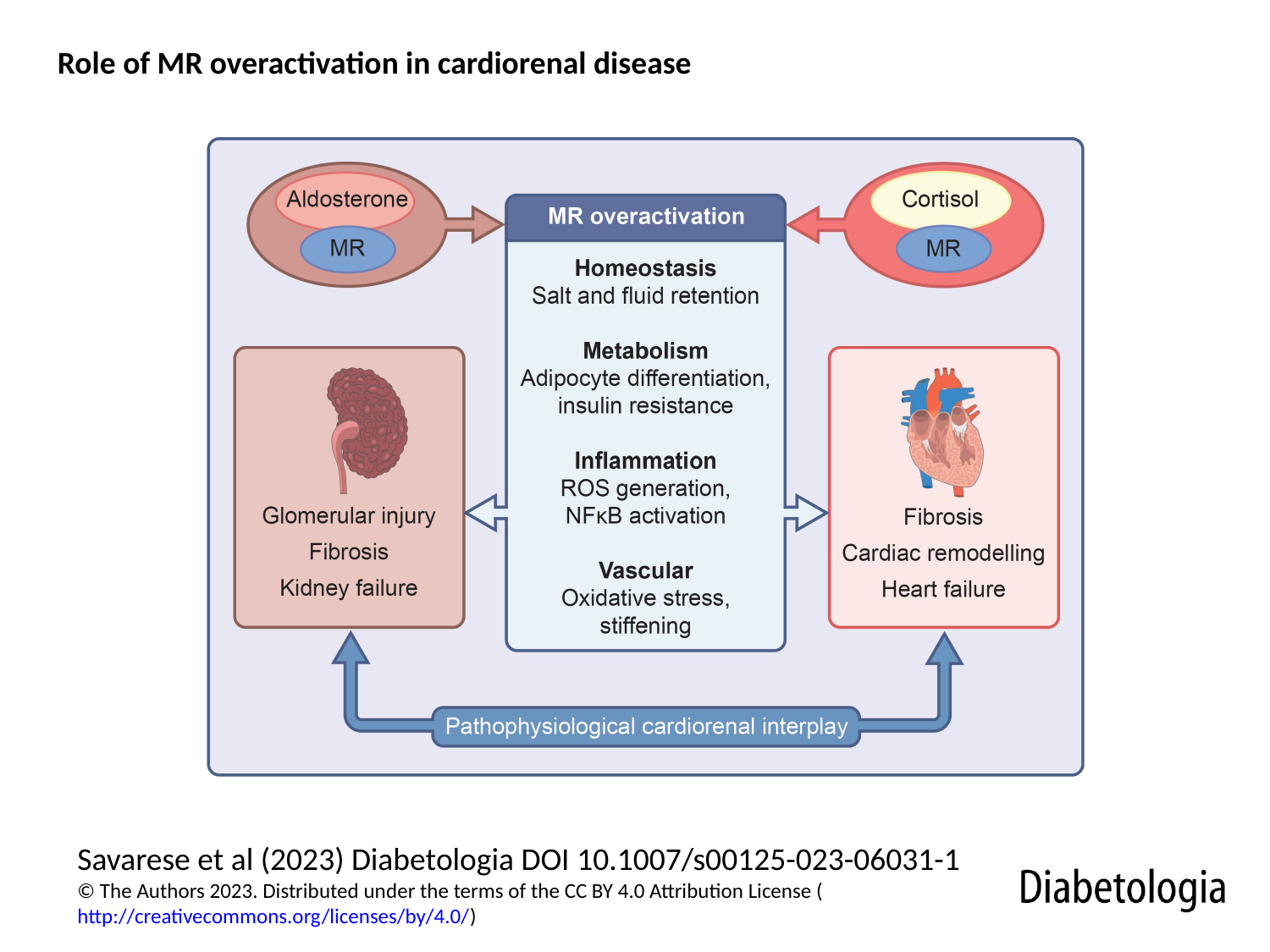

Role of MR overactivation in cardiorenal disease
Savarese et al (2023) Diabetologia DOI 10.1007/s00125-023-06031-1
© The Authors 2023. Distributed under the terms of the CC BY 4.0 Attribution License (http://creativecommons.org/licenses/by/4.0/)

## Slide 2
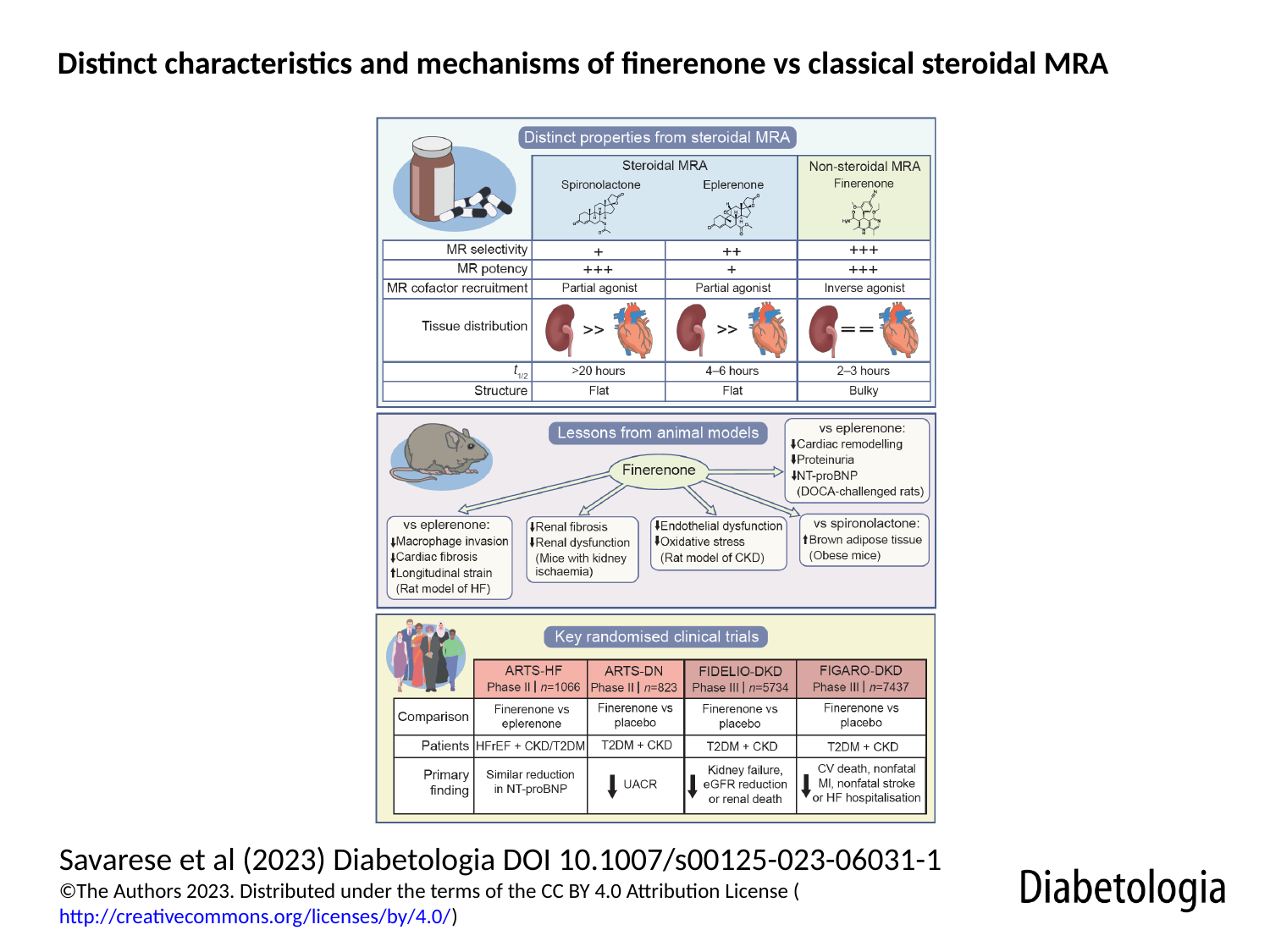

Distinct characteristics and mechanisms of finerenone vs classical steroidal MRA
Savarese et al (2023) Diabetologia DOI 10.1007/s00125-023-06031-1
©The Authors 2023. Distributed under the terms of the CC BY 4.0 Attribution License (http://creativecommons.org/licenses/by/4.0/)
